# Mercury(II) Complexes of Anionic *N*-Heterocyclic Carbene Ligands: Steric Effects of the Backbone Substituent

**DOI:** 10.3390/molecules25163741

**Published:** 2020-08-16

**Authors:** Chandrakanta Dash, Animesh Das, H. V. Rasika Dias

**Affiliations:** Department of Chemistry and Biochemistry, The University of Texas at Arlington, Arlington, TX 76019, USA; adas@iitg.ac.in

**Keywords:** ligands, *N*-heterocyclic carbene, mercury(II) complex, X-ray, T-shaped

## Abstract

Mercury(II) complexes (Me-maloNHC_Dipp_)HgCl (**1b**), (*t*-Bu-maloNHC_Dipp_)HgCl (**2b**) and (*t*-Bu-maloNHC_Dipp_)HgMe (**2c**) supported by anionic *N*-heterocyclic carbenes have been obtained in good yields from the reaction of the potassium salt of *N*-heterocyclic carbene ligand precursors and mercury(II) salts, HgCl_2_ and MeHgI. These molecules have been characterized by ^1^H-NMR, ^13^C-NMR and IR spectroscopy and elemental analysis. X-ray crystal structures of **1b** and **2b** are also presented. Interestingly, complex **1b** is polymeric {(Me-maloNHC_Dipp_)HgCl}_n_ in the solid state, as a result of inter-molecular Hg-O contacts, and features rare three coordinate mercury sites with a T-shaped arrangement, whereas the (*t*-Bu-maloNHC_Dipp_)HgCl (**2b**) is monomeric and has a linear, two-coordinate mercury center. The formation of T-shaped structure and the aggregation of complex **1b** is attributable to the reduced steric demand of the *N*-heterocyclic carbene ligand backbone substituent.

## 1. Introduction 

During the past three decades, considerable attention has been given to the chemistry of metal complexes of *N*-heterocyclic carbenes [1,2,3,4,5,6,7,8,9]. *N*-heterocyclic carbenes are versatile ligands with extensive applications in coordination chemistry and catalysis, as well as in bioinorganic chemistry [10,11,12,13,14,15,16,17]. Though traditional *N*-heterocyclic carbenes (NHCs) are neutral “L” type ligands, NHCs bearing tethered anionic donor sites (e.g., alkoxide, aryloxide, amido etc.) have also been reported, along with their metal complexes [18,19,20,21,22,23,24,25,26,27]. In contrast, *N*-heterocyclic carbene ligands with remote anionic functional groups/moieties within the heterocyclic ligand backbone have been less widely investigated [12,28,29]. Reactions of these anionic NHCs with transition metals afford corresponding zwitterionic complexes. Anionic, six-membered ring NHCs based on a malonate unit (maloNHC), introduced by Cesar, Lavigne and co-workers [30], are particularly interesting as they are attractive ligands to stabilize late transition metal ion complexes such as those involving Ag^I^, Au^I^, Rh^I^, Cu^I^ [30,31,32,33,34,35], as well as Fe^II^ [36].

**Figure 1:**
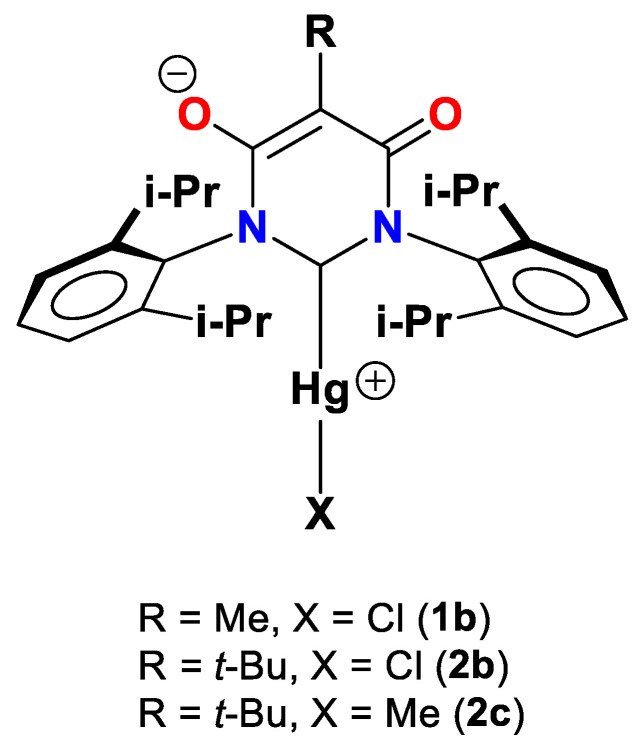
Mercury(II) complexes supported by anionic *N*-heterocyclic carbenes [Me-maloNHC_Dipp_]^−^ and [*t*-Bu-maloNHC_Dipp_]^−^.

The first known metal-NHC complex was a mercury(II) compound reported by Wanzlick and Schonherr in 1968 [37]. The mercury(II)-NHC complexes have played an important role in the development of *N*-heterocyclic carbene chemistry, but are still less explored compared to other metals of the d-block [38,39,40,41,42,43,44,45,46,47,48]. Furthermore, to our knowledge, mercury(II) complexes of anionic *N*-herterocyclic carbene ligands, such as maloNHC, have not been investigated. As a continuation of our interest in the NHCs and their metal chemistry [49,50,51,52,53,54,55,56,57,58,59,60], we have set out to probe the use of anionic *N*-heterocyclic carbene ligands in mercury chemistry. In particular, we describe the synthesis and spectroscopic data of three new mercury(II) complexes (Figure 1), (Me-maloNHC_Dipp_)HgCl (**1b**), (*t*-Bu-maloNHC_Dipp_)HgCl (**2b**) and (*t*-Bu-maloNHC_Dipp_)HgMe (**2c**), involving a bulkier maloNHC. We have also studied the effects of backbone substituent in heterocyclic ring (malonate unit), on the structure of the mercury complexes **1b** and **2b**. 

## 2. Results and Discussion

### Synthesis and Characterization

**Scheme 1:**
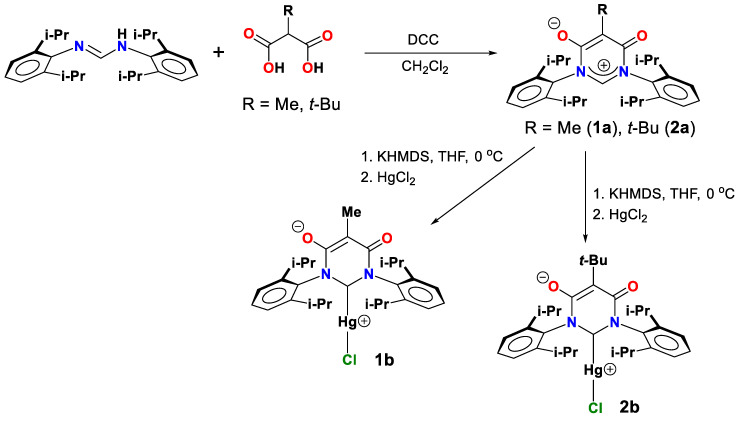
Synthesis of mercury(II) complexes **1b** and **2b** supported by anionic NHC.

The anionic, six-membered *N*-heterocyclic carbene ligand precursor **1a** has been reported earlier [32]. The anionic-NHC ligand precursors **1a** and **2a** were synthesized using the strategy reported by César et al. (from the condensation of *N,N’*-bis(2,6-diisopropylphenyl)formamidine and monosubstituted malonic acid) [30]. Moreover, ^1^H NMR spectrum of **2a** in CDCl_3_ at room temperature exhibits a signal corresponding to the NCHN proton at the δ 8.07 ppm (compare for **1a**: δ 8.06 ppm [32]). The mercury(II) complexes, (Me-maloNHC_Dipp_)HgCl (**1b**) and (*t*-Bu-maloNHC_Dipp_)HgCl (**2b**), were prepared from in situ generated anionic *N*-heterocyclic carbene ligand, by using KHMDS as the base and HgCl_2_ as the mercury source (Scheme 1). The complexes **1b** and **2b** are yellow crystalline solids, which were characterized by NMR, IR spectroscopy, elemental analysis and X-ray crystallography. They are air and light stable solids, and soluble in dichloromethane, THF and chloroform. The ^1^H NMR spectra of **1b** and **2b** in CDCl_3_ at room temperature showed the absence of NCHN resonance of the starting precursors (**1a** or **2a**). The ^13^C{^1^H} NMR spectrum showed the appearance of a highly downfield shifted mercury bound carbene carbon resonance at δ 180.9 and 179.9 ppm, for **1b** and **2b**, respectively. We did not observe Hg-C_carbene_ coupling in the ^13^C NMR spectrum, although large C_carbene_-Hg coupling in the range 2700–3300 Hz has been reported in the literature [61,62]. Yellow crystals of these molecules could be obtained from dichloromethane solution layering with hexane at −10 °C. 

**Figure 2:**
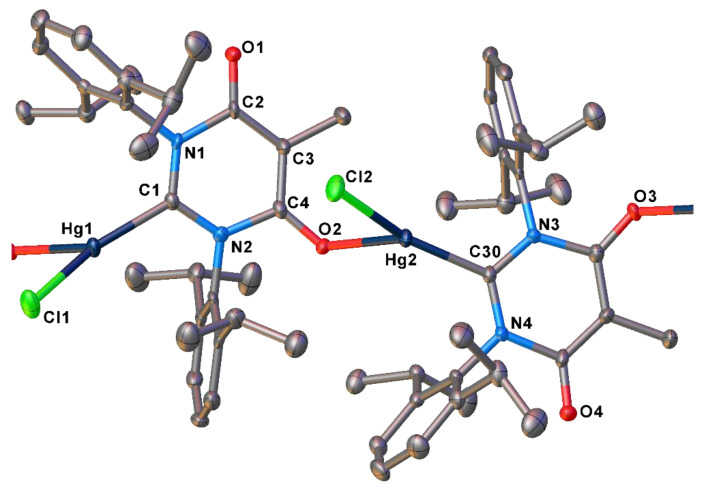
Molecular structure of (Me-maloNHC_Dipp_)HgCl (**1b**). Hydrogen atoms have been omitted for clarity. Selected bond distances (Å) and angles (°): Hg1-Cl1 2.3157(11), Hg1-O3 2.537(3), Hg1-C1 2.090(4), Hg2-Cl2 2.3152(11), Hg2-O2 2.529(3), Hg2-C30 2.092(4), O1-C2 1.228(5), O2-C4 1.262(5), O3-C31 1.256(5), O4-C33 1.227(5); Cl1-Hg1-O3^1^ 86.68(7); C1-Hg1-Cl1 157.34(12), C1-Hg1-O3 ^1^ 115.97(13), Cl2-Hg2-O2 86.52(7), C30-Hg2-Cl2 156.92(12), C30-Hg2-O2 116.55(13), C4-O2-Hg2 116.7(3), C31-O3-Hg1 ^2^ 117.3(3), where ^1^ +X, 1 + Y,+Z; ^2^ +X, −1 + Y, +Z.

**Figure 3:**
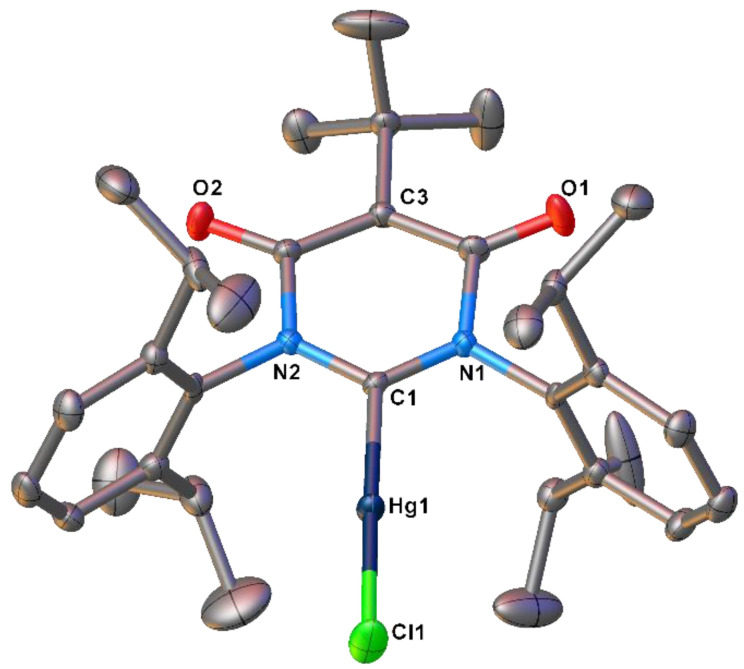
Molecular structure of (*t*-Bu-maloNHC_Dipp_)HgCl (**2b**). The second molecule in the asymmetric unit and hydrogen atoms have been omitted for clarity. Selected bond distances (Å) and angles (°): Molecule 1, Hg1-Cl1 2.2558(15), Hg1-C1 2.056(5), O1-C4 1.221(7), O2-C2 1.232(7); C1-Hg1-Cl1 177.50(15); Molecule 2 (not shown), Hg2-Cl2 2.235(2), Hg2-C33 2.062(5), O3-C34 1.229(7), O4-C36 1.220(7), C33-Hg2-Cl2 177.74(15).

The molecular structures of the complexes **1b** and **2b** are illustrated in Figure 2 and Figure 3. The complex **1b** crystallizes in P-1 space group with two chemically similar molecules of **1b** in the asymmetric unit. X-ray structure of **1b** shows the presence of three coordinated mercury atoms (and zig-zag polymeric chains resulting from bridging Hg-O contacts involving neighboring molecules) having a distorted T-shaped geometry with bond angles of 86.68(7)°, 157.34(12)°, 115.97(13)° at Hg1 and 86.52(7)°, 156.92(12)°, 116.55(13)° at Hg2. The inter-molecular Hg-O bond distances of 2.537(3) and 2.529(3) Å are shorter than the sum of van der Waals radii of the respective elements, which is 3.07 Å for an Hg^**…**^O interaction. For comparison, interactions of similar magnitude have been observed in the complex C_6_F_5_HgCl•DMSO [Hg-O = 2.542(2) Å], reported by the Gabbai group [63]. The average Hg-C_carbene_ bond distance in **1b** is 2.091(4) Å, which is in good agreement with the five membered imidazole based mercury carbene complexes, i.e., [(IDipp)HgCl_2_] [2.090(4) Å], [(IMes)HgCl_2_] [2.084(6) Å] [64]. The average Hg-Cl bond distance in **1b** (2.3154(11) Å) is comparable to the other three coordinated, T-shaped mercury complexes, namely, [2-(Me_2_NCH_2_)C_6_H_4_]HgCl [2.319(2) Å], C_6_F_5_HgCl•DMSO [2.322(2) Å] [63,65].

The complex (*t*-Bu-maloNHC_Dipp_)HgCl (**2b**) crystallizes in P-1 space group with two independent molecules of (*t*-Bu-maloNHC_Dipp_)HgCl in the asymmetric unit. In contrast to the complex **1b**, the mercury complex **2b** has a two-coordinate linear mercury center with C_carbene_-Hg-Cl bond angles of 177.50(15)° and 177.74(15)° for the two molecules. This is likely a result of having a bulky *t*-Bu moiety in the six-membered ring at the remote, apical carbon, in addition to the bulky N-aryl group, which inhibit the interaction of mercury atom with oxygen atom in an adjacent molecule. We should note here that electronic donor properties at the carbene center have been modulated by using electrophiles to interact with oxygen atoms of remote malonate group of Me-maloNHC_Mes_ and *t*-Bu-maloNHC_Mes_ in rhodium complexes [35]. The linear geometry at mercury of (*t*-Bu-maloNHC_Dipp_)HgCl is commonly found in Hg-NHC complexes involving five membered neutral NHCs. The average C_carbene_-Hg bond distance in **2b** (2.059(5) Å) is similar to those observed in the other mercury complexes reported in the literature. The Hg-Cl distances in **2b** 2.2558(15) and 2.235(2) Å are shorter than in those found in complex **1b** [2.3158(10) Å]. This is not surprising, as complex **1b** has three coordinate mercury sites, whereas they are two-coordinate in **2b**. 

**Scheme 2:**
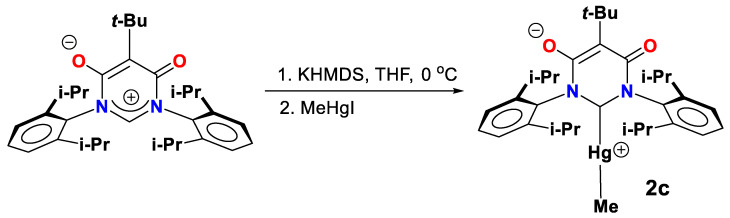
Synthesis of mercury(II) complex (*t*-Bu-maloNHC_Dipp_)HgMe (**2c**).

The mercury(II) complex, (*t*-Bu-maloNHC_Dipp_)HgMe (**2c**) was synthesized from a reaction between in situ generated anionic *N*-heterocyclic carbene ligand and MeHgI as the metal precursor (Scheme 2) in THF. It was isolated as a yellow colored solid in 70% yield. The ^13^C resonance of the mercury(II)-bound carbene carbon (NCN) of (*t*-Bu-maloNHC_Dipp_)HgMe (**2c**) in CDCl_3_ was observed as a singlet at δ 205.8 ppm. We have not attempted to obtain crystalline materials of **2c** for an X-ray crystallographic study.

## 3. Experimental Sections

### 3.1. Materials and General Methods

All manipulations were carried out under an atmosphere of dry nitrogen, using standard Schlenk techniques, or in a glove box. Solvents were purchased from commercial sources, purified using an Innovative Technology SPS-400 PureSolv solvent drying system (Innovative Technology, Inc., Galway, Ireland), degassed by the freeze-pump-thaw method twice prior to use. Glassware was oven-dried at 150 °C overnight. NMR spectra were recorded at 298 K on JEOL Eclipse 500 and 300 spectrometers (Jeol Ltd., Tokyo, Japan). NMR annotations used: br. = broad, d = doublet, m = multiplet, s = singlet, t = triplet, sept = septet. Infrared spectra were recorded on a JASCO FT-IR 410 spectrometer (Jasco, Tokyo, Japan) operating at 2 cm^−1^ spectral resolution. IR spectroscopic data were collected using KBr pellets. Herein, we use abbreviations based on IUPAC guidelines, that is, ν for frequency and ῡ for wavenumber. Elemental analyses were performed using a Perkin Elmer Series II CHNS/O analyzer. Methylmalonic acid, DCC were purchased from Sigma-Aldrich and used without further purification. *N,N’*-bis(2,6-diisopropylphenyl)formamidine [66] and *tert*-butylmalonic acid [67] were synthesized as reported, and anionic-NHC ligand precursor (**1a**) [32] was obtained via a modified literature procedure (noted below). The data of NMR spectrum can be found in the Supplementary Materials.

#### 3.1.1. Synthesis of 1,3-Bis(2,6-diisopropylphenyl)-5-methyl-6-oxo-6H-pyrimidinium-4-olate (**1a**)

A mixture of *N,N’*-bis(2,6-diisopropylphenyl)formamidine (1.81 g, 4.96 mmol) and *N,N’*-dicyclohexylcarbodiimide (DCC) (2.04 g, 9.92 mmol) in dichloromethane (ca. 35 mL) was placed in a 50 mL Schlenk flask. To this mixture, methylmalonic acid (0.586 g, 4.96 mmol) was added as a solid at room temperature. The reaction mixture was stirred for 5 h. The solution was filtered and the solvents were evaporated. The residue was purified by flash chromatography (SiO_2_, 30% EtOAc in hexane), to obtain a yellow crystalline solid (1.62 g, 73% yield). The analytical data agree with the reported values [32].

#### 3.1.2. Synthesis of 1,3-Bis(2,6-diisopropylphenyl)-5-tert-butyl-6-oxo-6H-pyrimidinium-4-olate (**2a**)

A mixture of *N,N’*-bis(2,6-diisopropylphenyl)formamidine (5.18 g, 14.2 mmol) and *N,N’*-dicyclohexylcarbodiimide (DCC) (5.86 g, 28.4 mmol) in dichloromethane (ca. 50 mL) was taken in a 100 mL Schlenk flask. To this mixture, *tert*-butylmalonic acid (2.27 g, 14.2 mmol) was then added as a solid at room temperature. The reaction mixture was stirred for 6 h. The solution was filtered and the solvents were evaporated. The residue was purified by chromatography (SiO_2_, 10% EtOAc in hexane) to give a yellow crystalline solid (4.71 g, 68% yield). Mp: 255–260 °C. ^1^H NMR (CDCl_3_, 500.16 MHz, 298 K): δ 8.07 (s, 1H, NCHN), 7.42 (t, 2H, ^3^J_HH_ = 8.0 Hz, C_6_H_3_), 7.25 (d, 4H, ^3^J_HH_ = 8.0 Hz, C_6_H_3_), 2.80 (sept, 4H, ^3^J_HH_ = 6.9 Hz, CH(CH_3_)_2_), 1.46 (s, 9H, C(CH_3_)_3_), 1.30 (d, 12H, ^3^J_HH_ = 6.9 Hz, CH(CH_3_)_2_), 1.20 (d, 12H, ^3^J_HH_ = 6.9 Hz, CH(CH_3_)_2_). ^13^C{^1^H} NMR (CDCl_3_, 125.77 MHz, 298 K): δ 158.3 (C-O), 146.9, 145.7, 132.5, 130.6, 124.2, 104.2, 34.5, 30.0, 29.2, 24.1, 23.9. IR (KBr) cm^−1^: 3068, 2991, 2856, 1926, 1778, 1760, 1687, 1598, 1581, 1462, 1425, 1387, 1364, 1351, 1327, 1304, 1282, 1258, 1218, 1183, 1149, 1107, 1059, 1018, 981, 937, 875, 803, 761, 741. Anal. Calcd. for C_32_H_44_N_2_O_2_: C, 78.65; H, 9.07; N, 5.73. Found: C, 79.01; H, 9.03; N, 6.04%.

#### 3.1.3. Synthesis of (Me-maloNHC_Dipp_)HgCl (**1b**)

To a solution of **1a** (0.491 g, 1.10 mmol) in THF (30 mL), KHMDS (0.5 M in toluene, 2.2 mL, 1.10 mmol, 1.0 equiv.) was added dropwise at 0 °C (ice bath). After 30 min at 0 °C, HgCl_2_ (0.299 g, 1.10 mmol) was added as a solid. The reaction mixture was stirred at room temperature for 6 h. The solvent was removed under vacuum, and the crude residue was dissolved in dichloromethane (30 mL). The mixture was filtered through a pad of Celite, and the filtrate was evaporated under vacuum to obtain a yellow solid. The compound was further washed with hexane and vacuum dried (0.405 g, 54% yield). Mp: 287 °C. ^1^H NMR (CDCl_3_, 500.16 MHz, 298 K): δ 7.53 (t, 2H, ^3^*J*_HH_ = 8.0 Hz, C_6_H_3_), 7.33 (d, 4H, ^3^*J*_HH_ = 8.0 Hz, C_6_H_3_), 2.80 (sept, 4H, ^3^*J*_HH_ = 6.9 Hz, CH(CH_3_)_2_), 2.02 (s, 3H, CH_3_), 1.27 (d, 12H, ^3^*J*_HH_ = 6.9 Hz, CH(CH_3_)_2_), 1.22 (d, 12H, ^3^*J*_HH_ = 6.9 Hz, CH(CH_3_)_2_). ^13^C{^1^H} NMR (CDCl_3_, 125.77 MHz, 298 K): δ 180.9 (Hg-NCN), 160.1 (C-O), 146.2, 136.7, 131.8, 125.4, 94.4, 29.1, 24.6, 24.4, 10.1. IR (KBr) cm^−1^: 2963, 2928, 2871, 1727, 1678, 1645, 1610, 1546, 1466, 1447, 1386, 1365, 1345, 1322, 1259, 1222, 1181, 1147, 1107, 1062, 1039, 934, 795, 755. Anal. Calcd. for C_29_H_37_N_2_O_2_HgCl•THF: C, 52.58; H, 6.02; N, 3.72. Found: C, 52.63; H, 5.81; N, 4.13%. 

#### 3.1.4. (*t*-Bu-maloNHC_Dipp_)HgCl (**2b**)

To a solution of **2a** (0.752 g, 1.54 mmol) in THF (30 mL), KHMDS (0.5 M in toluene, 3.4 mL, 1.70 mmol, 1.1 equiv.) was added dropwise at 0 °C (ice bath). After 30 min at 0 °C, HgCl_2_ (0.418 g, 1.54 mmol) was added as a solid. The reaction mixture was stirred at room temperature for 6 h. The solvent was removed under vacuum, and the crude residue was dissolved in dichloromethane (40 mL). The mixture was filtered through a pad of Celite, and the filtrate was evaporated under vacuum to obtain a yellow solid. The compound was further washed with hexane and vacuum dried (0.657 g, 59% yield). Mp: 250 °C. ^1^H NMR (CDCl_3_, 500.16 MHz, 298 K): δ 7.51 (t, 2H, ^3^*J*_HH_ = 8 Hz, C_6_H_3_), 7.33 (d, 4H, ^3^*J*_HH_ = 8 Hz, C_6_H_3_), 2.85 (sept, 4H, ^3^*J*_HH_ = 6.9 Hz, CH(CH_3_)_2_), 1.45 (s, 9H, C(CH_3_)_3_), 1.31 (d, 12H, ^3^*J*_HH_ = 6.9 Hz, CH(CH_3_)_2_), 1.26 (d, 12H, ^3^*J*_HH_ = 6.9 Hz, CH(CH_3_)_2_). ^13^C{^1^H} NMR (CDCl_3_, 125.77 MHz, 298 K): δ 179.9 (Hg-NCN), 159.1 (C-O), 146.1, 136.9, 131.6, 125.3, 104.1, 34.5, 30.0, 29.2, 24.4, 24.3. IR (KBr) cm^−1^: 3069, 2990, 2863, 1644, 1465, 1410, 1387, 1365, 1345, 1325, 1301, 1257, 1215, 1181, 1148, 1111, 1056, 1014, 979, 936, 879, 797, 759, 749. Anal. Calcd. for C_32_H_43_N_2_O_2_HgCl: C, 53.11; H, 5.99; N, 3.87. Found: C, 53.41; H, 6.52; N, 3.87%. 

#### 3.1.5. Synthesis of (*t*-Bu-maloNHC_Dipp_)HgMe (**2c**)

To a solution of 1,3-diisopropyl-5-*tert*-butyl-6-oxo-6H-pyrimidinium-4-olate (0.376 g, 0.77 mmol) in THF (20 mL), KHMDS (0.5 M in toluene, 1.92 mL, 0.96 mmol, 1.25 equiv.) was added dropwise at 0 °C (ice bath). After 30 min at 0 °C, MeHgI (0.264 g, 0.77 mmol) was added as a solid. The reaction mixture was stirred at room temperature for 12 h. The solvent was removed under vacuum, and the crude residue was dissolved in dichloromethane (20 mL). The mixture was filtered through a pad of Celite, and the filtrate was evaporated under vacuum to obtain a yellow solid. The compound was washed with hexane and vacuum dried (0.380 g, 70% yield). ^1^H NMR (CDCl_3_, 500.16 MHz, 298 K): δ 7.43 (t, 2H, ^3^*J*_HH_ = 8 Hz, C_6_H_3_), 7.27 (d, 4H, ^3^*J*_HH_ = 7.5 Hz, C_6_H_3_), 2.88 (sept, 4H, ^3^*J*_HH_ = 6.9 Hz, CH(CH_3_)_2_), 1.45 (s, 9H, C(CH_3_)_3_), 1.29 (d, 12H, ^3^*J*_HH_ = 6.9 Hz, CH(CH_3_)_2_), 1.21 (d, 12H, ^3^*J*_HH_ = 6.9 Hz, CH(CH_3_)_2_), 0.21 (s, 3H, CH_3_). ^13^C{^1^H} NMR (CDCl_3_, 125.77 MHz, 298 K): δ 205.8 (HgNCN), 160.3 (*C*-O), 146.3, 136.2, 130.5, 124.6, 104.0, 34.5, 30.2, 29.0, 24.3, 24.2, 4.9 (HgCH_3_). Anal. Calcd. for C_33_H_46_N_2_O_2_Hg: C, 56.35; H, 6.59; N, 3.98. Found: C, 55.98; H, 6.37; N, 4.11%.

### 3.2. Crystallographic Data Collection and Refinement 

A suitable crystal covered with a layer of hydrocarbon/Paratone-*N* oil was selected and mounted with in a Cryo-loop, and immediately placed in the low-temperature nitrogen stream. Diffraction data were collected at T = 100(2) K. The data sets were collected on a Bruker SMART APEX II CCD detector diffractometer with graphite monochromated Mo Kα radiation (λ = 0.71073 Å). The cell parameters were obtained from a least-squares refinement of the spots (from 60 collected frames), using the SMART program. Intensity data were processed using the Bruker ApexII program suite. Absorption corrections were applied by using SADABS. Initial atomic positions were located by direct methods using XS, and the structures of the compounds were refined by the least-squares method using SHELXL [68]. All the hydrogen atoms were refined anisotropically. Hydrogen positions were input and refined in a riding manner, along with the attached carbons. X-ray structural figures were generated using Olex2 [69]. The CCDC 2019022–2019023 contains the supplementary crystallographic data. These data can be obtained free of charge via http://www.ccdc.cam.ac.uk/conts/retrieving.html or from the Cambridge Crystallographic Data Centre (CCDC) (12 Union Road, Cambridge, CB2 1EZ, UK). 

Crystal data for (Me-maloNHC_Dipp_)HgCl (**1b**): C_58_H_74_Cl_2_Hg_2_N_4_O_4_ (M = 1363.29 g/mol): triclinic, space group *P*$\bar{1}$ (no. 2), *a* = 10.7845(8) Å, *b* = 13.9152(11) Å, *c* = 19.1697(15) Å, *α* = 106.9910(10)°, *β* = 91.2040(10)°, *γ* = 90.0910(10)°, V = 2750.5(4) Å^3^, Z = 2, T = 100.15 K, μ(MoKα) = 5.721 mm^−1^, *Dcalc* = 1.646 g/cm^3^, 22,195 reflections measured (3.06° ≤ 2Θ ≤ 50.996°), 10,213 unique (R_int_ = 0.0257, R_sigma_ = 0.0369), which were used in all calculations. The final R_1_ was 0.0313 (I > 2σ(I)) and *wR*_2_ was 0.0797 (all data).

Crystal data for (*t*-Bu-maloNHC_Dipp_)HgCl (**2b**): C_32_H_43_ClHgN_2_O_2_ (M = 723.72 g/mol): triclinic, space group *P*$\bar{1}$ (no. 2), *a* = 10.9912(9) Å, *b* = 14.9118(13) Å, *c* = 19.9787(17) Å, *α* = 99.9700(10)°, *β* = 98.6110(10)°, *γ* = 90.2430(10)°, V = 3187.1(5) Å^3^, Z = 4, T = 100.15 K, μ(MoKα) = 4.942 mm^−1^, *Dcalc* = 1.508 g/cm^3^, 27,908 reflections measured (2.774° ≤ 2Θ ≤ 53.464°), 13,462 unique (R_int_ = 0.0260, R_sigma_ = 0.0407), which were used in all calculations. The final R_1_ was 0.0405 (I > 2σ(I)) and *wR*_2_ was 0.1067 (all data).

## 4. Conclusions

In summary, we report the isolation of three new mercury(II) complexes supported by anionic maloNHC ligands. Solid state structures of **1b** and **2b** illustrate the effects of backbone substituent in maloNHC, supplemented by the N-aryl substituent steric demands have on the aggregation of these mercury complexes. The (Me-maloNHC_Dipp_)HgCl (**1b**) bearing methyl substituent in the malonate unit forms a polymeric mercury(II) complex, with a rare, distorted T-shaped structure, whereas (*t*-Bu-maloNHC_Dipp_)HgCl (**2b**) having a *t*-butyl substituent on the ligand backbone, remains monomeric. The [*t*-Bu-maloNHC_Dipp_]^−^ is a sterically demanding and useful supporting ligand to stabilize low-coordinate metal complexes. 

## Supplementary Materials

The following are available online. Crystal data, structure refinement, bond distances and angles for (Me-maloNHCDipp)HgCl (**1b**) and (*t*-Bu-maloNHCDipp)HgCl (**2b**), additional figures, and NMR spectra of new molecules.
