# Supplementary material for: Mercury(II) Complexes of Anionic N-Heterocyclic Carbene Ligands: Steric Effects of the Backbone Substituent
Source: Molecules. 2020 Aug 16;25(16):3741. doi: 10.3390/molecules25163741 (PMC7464600; doi:10.3390/molecules25163741)
Supplement: Supplementary file 1 [file molecules-25-03741-s001.pdf]

Supporting Information

Mercury(II) Complexes of Anionic N-Heterocyclic Carbene Ligands: Steric Effects of the Backbone Substituent

Chandrakanta Dash,*,† Animesh Das,‡ H. V. Rasika Dias*

Department of Chemistry and Biochemistry, The University of Texas at Arlington, Arlington, Texas 76019, USA

Correspondence: ckdash@curaj.ac.in; dias@uta.edu

† Current address: Department of Chemistry, School of Chemical Sciences and Pharmacy, Central University of Rajasthan, Bandar Sindri, Ajmer-305817, Rajasthan, India

‡Current address: Department of Chemistry, Indian Institute of Technology Guwahati, Guwahati-781039, Assam, India

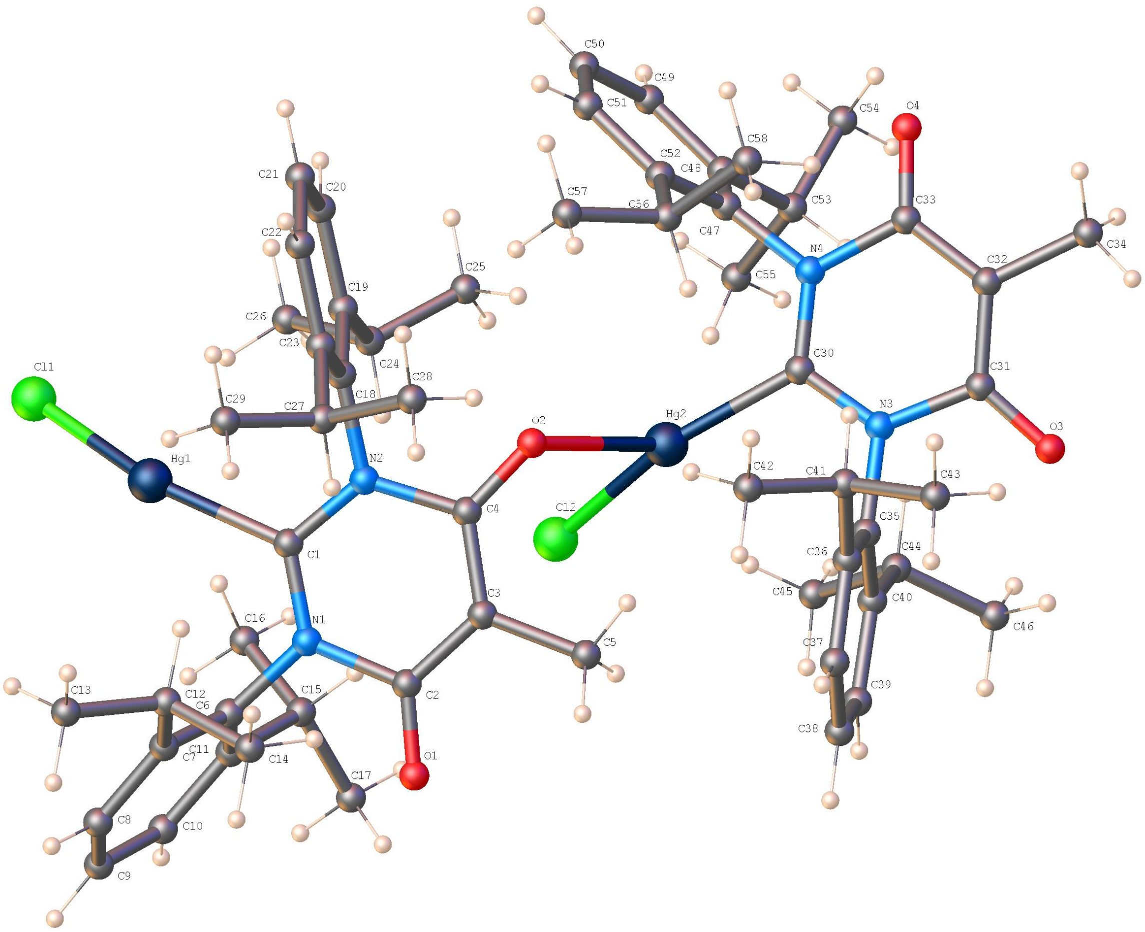

Figure S1. Asymmetric unit and atom labelling scheme of (Me-maloNHC$_{Dipp}$)HgCl (**1b**)

Table S1. Crystal data and structure refinement for (Me-maloNHC$_{Dipp}$)HgCl (**1b**).

Identification code	Hgcif-both
Empirical formula	$C_{58}H_{74}Cl_2Hg_2N_4O_4$
Formula weight	1363.29
Temperature/K	100.15
Crystal system	triclinic
Space group	P-1
a/Å	10.7845(8)
b/Å	13.9152(11)
c/Å	19.1697(15)
α/°	106.9910(10)
β/°	91.2040(10)
γ/°	90.0910(10)
Volume/Å^3	2750.5(4)
Z	2
ρ_{calc}g/cm^3	1.646

μ/mm^{-1}	5.721
F(000)	1352.0
Crystal size/mm^3	0.13 × 0.08 × 0.07
Radiation	MoKα (λ = 0.71073)
2Θ range for data collection/°	3.06 to 50.996
Index ranges	-13 ≤ h ≤ 13, -16 ≤ k ≤ 16, -23 ≤ l ≤ 23
Reflections collected	22195
Independent reflections	10213 [R_{int} = 0.0257, R_{sigma} = 0.0369]
Data/restraints/parameters	10213/0/649
Goodness-of-fit on F^2	1.036
Final R indexes [I>=2σ (I)]	R_1 = 0.0313, wR_2 = 0.0753
Final R indexes [all data]	R_1 = 0.0399, wR_2 = 0.0797

Table S2. Bond Lengths for (Me-maloNHC$_{Dipp}$)HgCl (**1b**).

Atom	Atom	Length/Å	Atom	Atom	Length/Å
Hg1	Cl1	2.3157(11)	C18	C23	1.400(6)
Hg1	O3^1	2.537(3)	C19	C20	1.401(6)
Hg1	C1	2.090(4)	C19	C24	1.520(7)
Hg2	Cl2	2.3152(11)	C20	C21	1.361(7)
Hg2	O2	2.529(3)	C21	C22	1.389(7)
Hg2	C30	2.092(4)	C22	C23	1.391(6)
O1	C2	1.228(5)	C23	C27	1.529(6)
O2	C4	1.262(5)	C24	C25	1.529(6)
O3	C31	1.256(5)	C24	C26	1.531(7)
O4	C33	1.227(5)	C27	C28	1.527(6)
N1	C1	1.329(5)	C27	C29	1.522(6)
N1	C2	1.472(5)	C31	C32	1.393(6)
N1	C6	1.454(5)	C32	C33	1.393(6)
N2	C1	1.344(5)	C32	C34	1.511(6)
N2	C4	1.444(5)	C35	C36	1.402(6)
N2	C18	1.455(5)	C35	C40	1.407(6)
N3	C30	1.335(5)	C36	C37	1.401(6)
N3	C31	1.452(5)	C36	C41	1.517(6)
N3	C35	1.458(5)	C37	C38	1.381(6)
N4	C30	1.326(5)	C38	C39	1.368(7)
N4	C33	1.469(5)	C39	C40	1.404(6)
N4	C47	1.460(5)	C40	C44	1.509(7)
C2	C3	1.397(5)	C41	C42	1.519(6)
C3	C4	1.391(6)	C41	C43	1.535(7)
C3	C5	1.500(6)	C44	C45	1.538(7)
C6	C7	1.399(6)	C44	C46	1.530(6)
C6	C11	1.394(6)	C47	C48	1.403(6)

C7	C8	1.386(6)	C47	C52	1.399(6)
C7	C12	1.523(6)	C48	C49	1.394(6)
C8	C9	1.386(6)	C48	C53	1.508(6)
C9	C10	1.378(6)	C49	C50	1.380(6)
C10	C11	1.395(6)	C50	C51	1.392(6)
C11	C15	1.526(6)	C51	C52	1.392(6)
C12	C13	1.527(6)	C52	C56	1.517(6)
C12	C14	1.528(6)	C53	C54	1.533(6)
C15	C16	1.528(7)	C53	C55	1.541(6)
C15	C17	1.539(7)	C56	C57	1.529(6)
C18	C19	1.396(6)	C56	C58	1.539(6)

[1]+X,1+Y,+Z

Table S3. Bond Angles for (Me-maloNHC$_{Dipp}$)HgCl (**1b**).

Atom	Atom	Atom	Angle/°	Atom	Atom	Atom	Angle/°
Cl1	Hg1	O3[1]	86.68(7)	C21	C22	C23	120.7(5)
C1	Hg1	Cl1	157.34(12)	C18	C23	C27	123.0(4)
C1	Hg1	O3[1]	115.97(13)	C22	C23	C18	116.6(4)
Cl2	Hg2	O2	86.52(7)	C22	C23	C27	120.4(4)
C30	Hg2	Cl2	156.92(12)	C19	C24	C25	111.7(4)
C30	Hg2	O2	116.55(13)	C19	C24	C26	113.4(4)
C4	O2	Hg2	116.7(3)	C25	C24	C26	107.8(4)
C31	O3	Hg1[2]	117.3(3)	C28	C27	C23	111.4(4)
C1	N1	C2	123.0(3)	C29	C27	C23	113.4(4)
C1	N1	C6	121.4(3)	C29	C27	C28	110.8(4)
C6	N1	C2	115.6(3)	N3	C30	Hg2	117.2(3)
C1	N2	C4	123.7(3)	N4	C30	Hg2	123.5(3)
C1	N2	C18	118.5(3)	N4	C30	N3	119.1(3)
C4	N2	C18	117.5(3)	O3	C31	N3	116.7(4)
C30	N3	C31	123.6(3)	O3	C31	C32	128.0(4)
C30	N3	C35	119.1(3)	C32	C31	N3	115.3(3)
C31	N3	C35	117.1(3)	C31	C32	C34	119.4(4)
C30	N4	C33	123.3(3)	C33	C32	C31	123.0(4)
C30	N4	C47	121.5(3)	C33	C32	C34	117.4(4)
C47	N4	C33	115.2(3)	O4	C33	N4	117.0(4)
N1	C1	Hg1	123.5(3)	O4	C33	C32	127.7(4)
N1	C1	N2	118.8(4)	C32	C33	N4	115.3(3)
N2	C1	Hg1	117.4(3)	C36	C35	N3	116.2(4)
O1	C2	N1	116.6(3)	C36	C35	C40	125.3(4)

O1	C2	C3	127.7(4)	C40	C35	N3	118.5(4)
C3	C2	N1	115.7(3)	C35	C36	C41	123.0(4)
C2	C3	C5	117.3(4)	C37	C36	C35	115.5(4)
C4	C3	C2	122.4(4)	C37	C36	C41	121.5(4)
C4	C3	C5	120.0(4)	C38	C37	C36	121.3(4)
O2	C4	N2	116.7(4)	C39	C38	C37	121.1(4)
O2	C4	C3	127.4(4)	C38	C39	C40	121.8(4)
C3	C4	N2	115.9(4)	C35	C40	C44	123.0(4)
C7	C6	N1	118.7(4)	C39	C40	C35	115.0(4)
C11	C6	N1	117.4(4)	C39	C40	C44	121.9(4)
C11	C6	C7	123.6(4)	C36	C41	C42	112.8(4)
C6	C7	C12	121.7(4)	C36	C41	C43	111.4(4)
C8	C7	C6	116.7(4)	C42	C41	C43	109.9(4)
C8	C7	C12	121.6(4)	C40	C44	C45	113.2(4)
C7	C8	C9	121.4(4)	C40	C44	C46	110.9(4)
C10	C9	C8	120.4(4)	C46	C44	C45	108.4(4)
C9	C10	C11	120.8(4)	C48	C47	N4	117.1(4)
C6	C11	C10	117.1(4)	C52	C47	N4	118.8(4)
C6	C11	C15	122.0(4)	C52	C47	C48	123.9(4)
C10	C11	C15	120.9(4)	C47	C48	C53	122.7(4)
C7	C12	C13	112.6(4)	C49	C48	C47	116.9(4)
C7	C12	C14	111.8(3)	C49	C48	C53	120.4(4)
C13	C12	C14	109.9(4)	C50	C49	C48	120.6(4)
C11	C15	C16	109.9(4)	C49	C50	C51	121.2(4)
C11	C15	C17	111.7(4)	C50	C51	C52	120.6(4)
C16	C15	C17	111.4(4)	C47	C52	C56	121.8(4)
C19	C18	N2	119.2(4)	C51	C52	C47	116.8(4)
C19	C18	C23	124.3(4)	C51	C52	C56	121.4(4)
C23	C18	N2	116.5(4)	C48	C53	C54	111.9(4)
C18	C19	C20	115.8(4)	C48	C53	C55	110.2(4)
C18	C19	C24	123.4(4)	C54	C53	C55	111.2(4)
C20	C19	C24	120.7(4)	C52	C56	C57	112.7(4)
C21	C20	C19	121.8(4)	C52	C56	C58	111.5(3)
C20	C21	C22	120.8(4)	C57	C56	C58	109.5(4)

[1]+X,1+Y,+Z; [2]+X,-1+Y,+Z

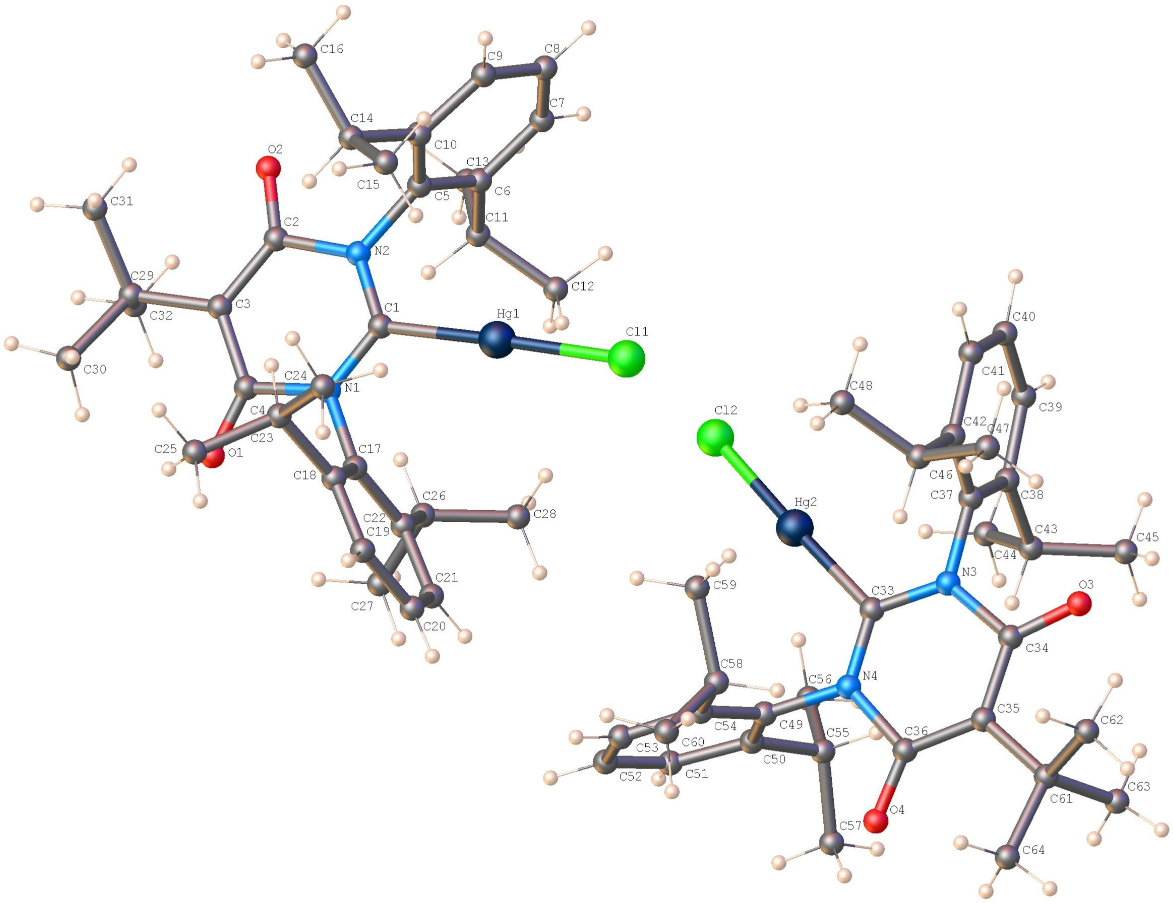

Figure S2. Asymmetric unit and atom labelling scheme of (*t*-Bu-maloNHC$_{Dipp}$)HgCl (**2b**)

Table S4. Crystal data and structure refinement for (*t*-Bu-maloNHC$_{Dipp}$)HgCl (2b)

Empirical formula	$C_{32}H_{43}ClHgN_2O_2$
Formula weight	723.72
Temperature/K	100.15
Crystal system	triclinic
Space group	P-1
a/Å	10.9912(9)
b/Å	14.9118(13)
c/Å	19.9787(17)
α/°	99.9700(10)
β/°	98.6110(10)
γ/°	90.2430(10)
Volume/Å^3	3187.1(5)

Z	4
ρ_{calc}g/cm^3	1.508
μ/mm^{-1}	4.942
F(000)	1448.0
Crystal size/mm^3	0.19 × 0.1 × 0.02
Radiation	MoKα (λ = 0.71073)
2Θ range for data collection/°	2.774 to 53.464
Index ranges	-13 ≤ h ≤ 13, -18 ≤ k ≤ 18, -25 ≤ l ≤ 25
Reflections collected	27908
Independent reflections	13462 [R_{int} = 0.0260, R_{sigma} = 0.0407]
Data/restraints/parameters	13462/0/707
Goodness-of-fit on F^2	1.041
Final R indexes [I>=2σ (I)]	R_1 = 0.0405, wR_2 = 0.1003
Final R indexes [all data]	R_1 = 0.0550, wR_2 = 0.1067

Table S5. Bond Lengths for (*t*-Bu-maloNHC$_{Dipp}$)HgCl (2b)

Atom	Atom	Length/Å	Atom	Atom	Length/Å
Hg1	Cl1	2.2558(15)	Hg2	Cl2	2.235(2)
Hg1	C1	2.056(5)	Hg2	C33	2.062(5)
O1	C4	1.221(7)	O3	C34	1.229(7)
O2	C2	1.232(7)	O4	C36	1.220(7)
N1	C1	1.318(7)	N3	C33	1.322(7)
N1	C4	1.475(7)	N3	C34	1.480(7)
N1	C17	1.453(7)	N3	C37	1.442(7)
N2	C1	1.320(7)	N4	C33	1.314(7)
N2	C2	1.470(7)	N4	C36	1.483(7)
N2	C5	1.464(7)	N4	C49	1.463(6)
C2	C3	1.410(8)	C34	C35	1.419(8)
C3	C4	1.413(8)	C35	C36	1.417(7)
C3	C29	1.546(7)	C35	C61	1.535(7)
C5	C6	1.401(8)	C37	C38	1.402(8)
C5	C10	1.382(8)	C37	C42	1.398(8)
C6	C7	1.396(8)	C38	C39	1.395(9)
C6	C11	1.517(8)	C38	C43	1.527(10)
C7	C8	1.380(9)	C39	C40	1.387(11)
C8	C9	1.382(9)	C40	C41	1.378(10)
C9	C10	1.401(8)	C41	C42	1.400(9)
C10	C14	1.523(8)	C42	C46	1.502(9)
C11	C12	1.518(10)	C43	C44	1.528(10)
C11	C13	1.515(10)	C43	C45	1.513(11)
C14	C15	1.509(10)	C46	C47	1.536(10)
C14	C16	1.493(10)	C46	C48	1.538(9)

C17	C18	1.412(8)	C49	C50	1.403(8)
C17	C22	1.395(8)	C49	C54	1.398(8)
C18	C19	1.388(8)	C50	C51	1.392(8)
C18	C23	1.522(8)	C50	C55	1.524(8)
C19	C20	1.385(8)	C51	C52	1.379(8)
C20	C21	1.382(8)	C52	C53	1.387(8)
C21	C22	1.398(8)	C53	C54	1.397(7)
C22	C26	1.516(8)	C54	C58	1.512(8)
C23	C24	1.522(9)	C55	C56	1.520(9)
C23	C25	1.490(9)	C55	C57	1.515(9)
C26	C27	1.527(9)	C58	C59	1.544(8)
C26	C28	1.533(9)	C58	C60	1.530(8)
C29	C30	1.536(9)	C61	C62	1.551(8)
C29	C31	1.533(8)	C61	C63	1.542(8)
C29	C32	1.513(9)	C61	C64	1.534(8)

Table S6. Bond Angles for (*t*-Bu-maloNHC$_{Dipp}$)HgCl (2b)

Atom	Atom	Atom	Angle/°	Atom	Atom	Atom	Angle/°
C1	Hg1	Cl1	177.50(15)	C33	Hg2	Cl2	177.74(15)
C1	N1	C4	123.3(4)	C33	N3	C34	122.8(5)
C1	N1	C17	117.4(4)	C33	N3	C37	118.7(5)
C17	N1	C4	119.3(4)	C37	N3	C34	118.5(4)
C1	N2	C2	123.3(5)	C33	N4	C36	123.4(4)
C1	N2	C5	117.2(4)	C33	N4	C49	118.8(4)
C5	N2	C2	119.5(4)	C49	N4	C36	117.7(4)
N1	C1	Hg1	120.0(4)	N3	C33	Hg2	117.8(4)
N1	C1	N2	120.0(5)	N4	C33	Hg2	121.5(4)
N2	C1	Hg1	119.9(4)	N4	C33	N3	120.6(5)
O2	C2	N2	114.0(5)	O3	C34	N3	114.2(5)
O2	C2	C3	130.3(5)	O3	C34	C35	129.8(5)
C3	C2	N2	115.7(5)	C35	C34	N3	116.1(5)
C2	C3	C4	121.2(5)	C34	C35	C61	117.3(5)
C2	C3	C29	118.6(5)	C36	C35	C34	120.9(5)
C4	C3	C29	119.7(5)	C36	C35	C61	121.4(5)
O1	C4	N1	114.6(5)	O4	C36	N4	113.9(5)
O1	C4	C3	130.4(5)	O4	C36	C35	130.8(5)
C3	C4	N1	115.0(5)	C35	C36	N4	115.3(5)
C6	C5	N2	117.7(5)	C38	C37	N3	118.8(5)
C10	C5	N2	118.2(5)	C42	C37	N3	118.3(5)
C10	C5	C6	123.9(5)	C42	C37	C38	122.9(5)

C5	C6	C11	123.9(5)	C37	C38	C43	123.0(5)
C7	C6	C5	116.3(5)	C39	C38	C37	117.6(6)
C7	C6	C11	119.8(5)	C39	C38	C43	119.4(6)
C8	C7	C6	122.0(6)	C40	C39	C38	120.7(7)
C7	C8	C9	119.5(5)	C41	C40	C39	120.5(6)
C8	C9	C10	121.4(6)	C40	C41	C42	121.1(6)
C5	C10	C9	117.0(5)	C37	C42	C41	117.2(6)
C5	C10	C14	123.1(5)	C37	C42	C46	122.1(5)
C9	C10	C14	119.9(5)	C41	C42	C46	120.7(6)
C6	C11	C12	112.1(6)	C38	C43	C44	110.8(7)
C13	C11	C6	110.8(5)	C45	C43	C38	110.6(6)
C13	C11	C12	110.8(6)	C45	C43	C44	111.6(6)
C15	C14	C10	112.0(6)	C42	C46	C47	110.3(5)
C16	C14	C10	111.2(6)	C42	C46	C48	112.5(7)
C16	C14	C15	110.5(7)	C47	C46	C48	109.9(6)
C18	C17	N1	117.6(5)	C50	C49	N4	118.3(5)
C22	C17	N1	119.2(5)	C54	C49	N4	118.5(5)
C22	C17	C18	123.1(5)	C54	C49	C50	123.2(5)
C17	C18	C23	122.3(5)	C49	C50	C55	123.3(5)
C19	C18	C17	116.9(5)	C51	C50	C49	116.7(5)
C19	C18	C23	120.8(5)	C51	C50	C55	120.0(5)
C20	C19	C18	121.4(5)	C52	C51	C50	121.8(5)
C21	C20	C19	120.3(5)	C51	C52	C53	120.1(5)
C20	C21	C22	121.1(5)	C52	C53	C54	120.9(5)
C17	C22	C21	117.1(5)	C49	C54	C58	121.9(5)
C17	C22	C26	122.9(5)	C53	C54	C49	117.3(5)
C21	C22	C26	119.9(5)	C53	C54	C58	120.9(5)
C24	C23	C18	110.5(5)	C56	C55	C50	112.1(5)
C25	C23	C18	110.5(5)	C57	C55	C50	109.5(5)
C25	C23	C24	112.5(8)	C57	C55	C56	110.2(5)
C22	C26	C27	110.0(5)	C54	C58	C59	111.2(5)
C22	C26	C28	111.7(5)	C54	C58	C60	113.0(5)
C27	C26	C28	110.2(5)	C60	C58	C59	109.1(5)
C30	C29	C3	111.3(5)	C35	C61	C62	111.4(5)
C31	C29	C3	111.8(5)	C35	C61	C63	109.3(5)
C31	C29	C30	105.5(5)	C63	C61	C62	108.2(5)
C32	C29	C3	109.5(5)	C64	C61	C35	113.8(5)
C32	C29	C30	109.8(6)	C64	C61	C62	105.8(5)
C32	C29	C31	108.9(6)	C64	C61	C63	108.1(5)

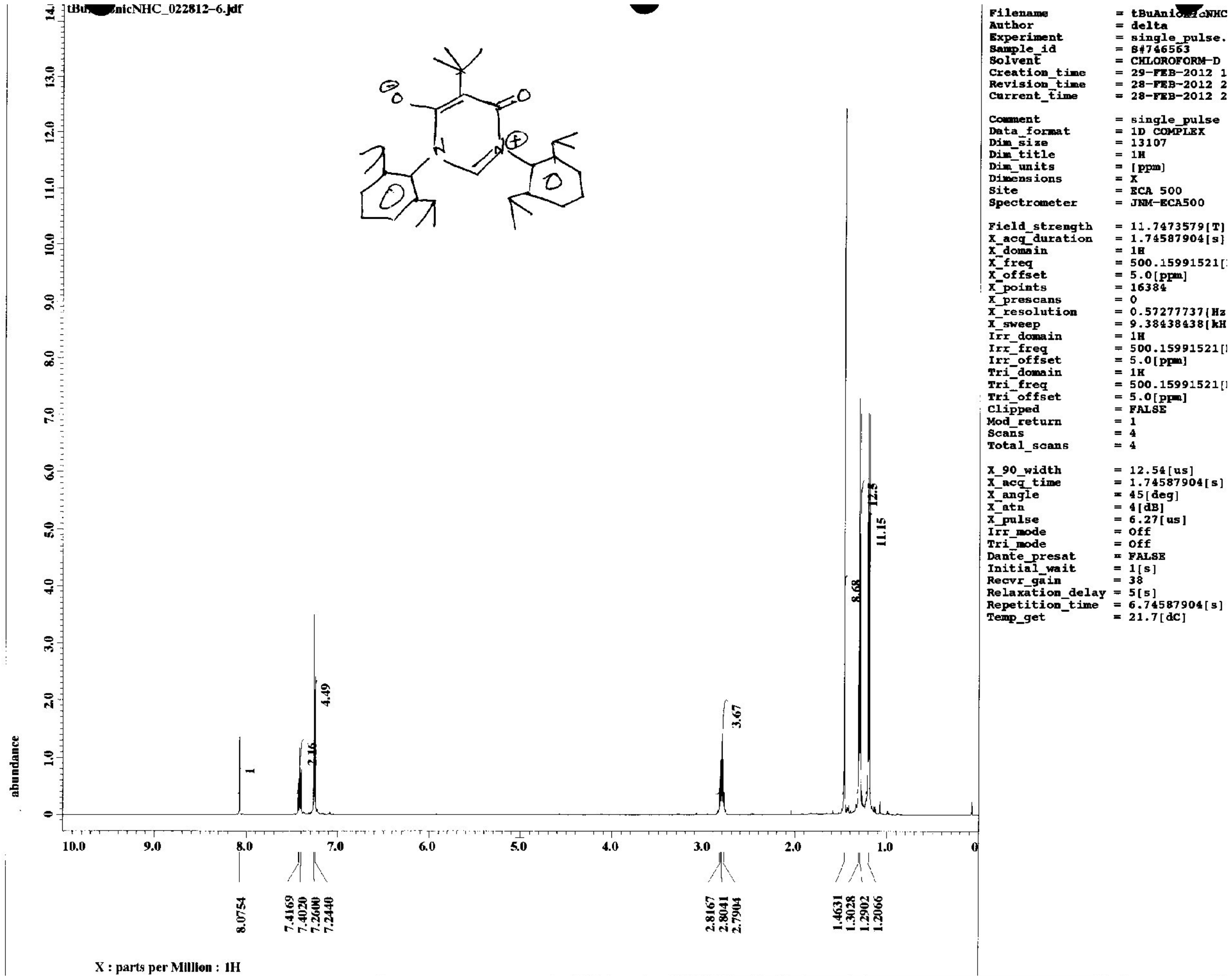

Figure S3. ^{1}H NMR spectrum of [*t*-Bu-maloNHC$_{Dipp}$]H (**2a**) in $CDCl_3$ at room temperature

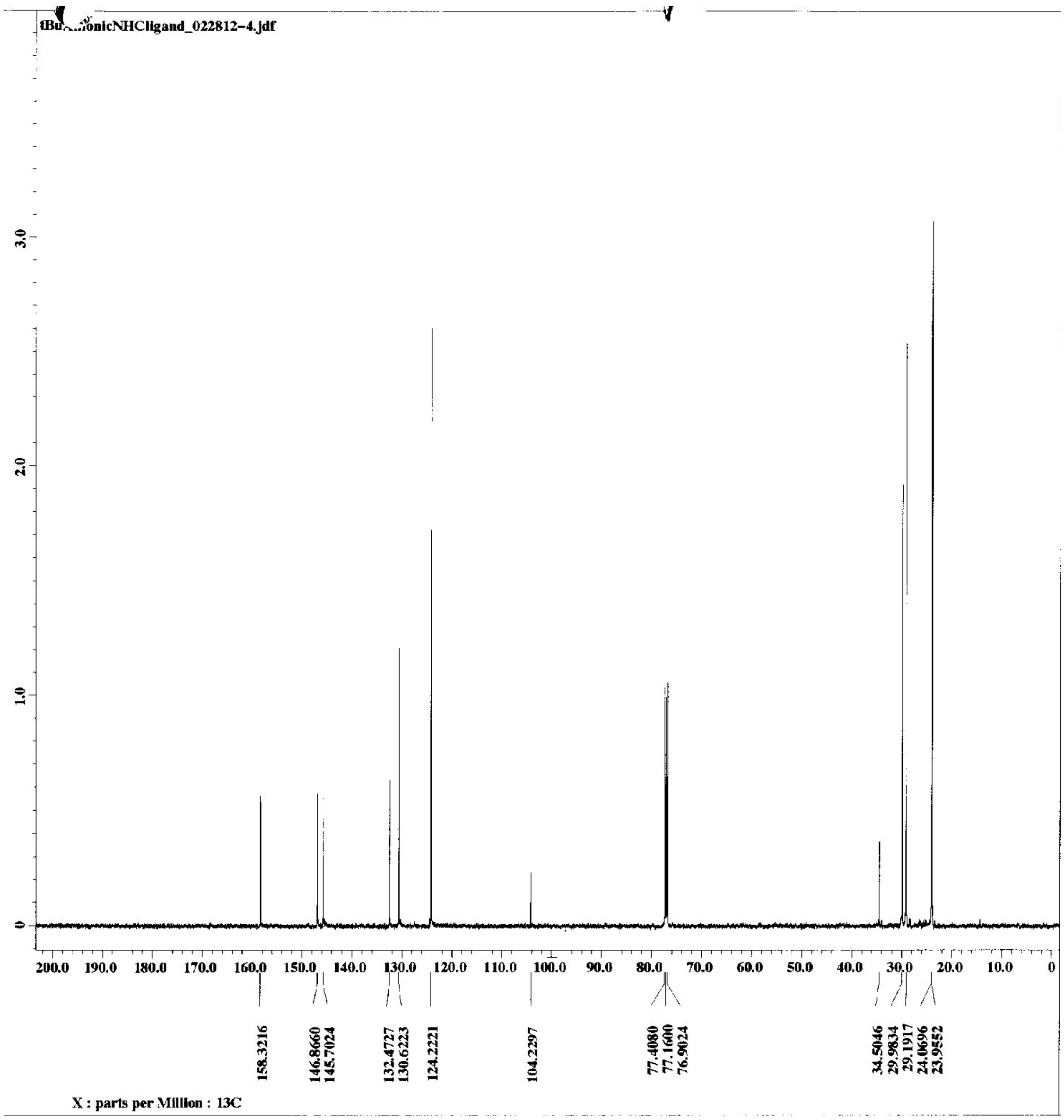

Figure S4. $^{13}C\{^{1}H\}$ NMR spectrum of [*t*-Bu-maloNHC$_{Dipp}$]H (**2a**) in $CDCl_3$ at room temperature

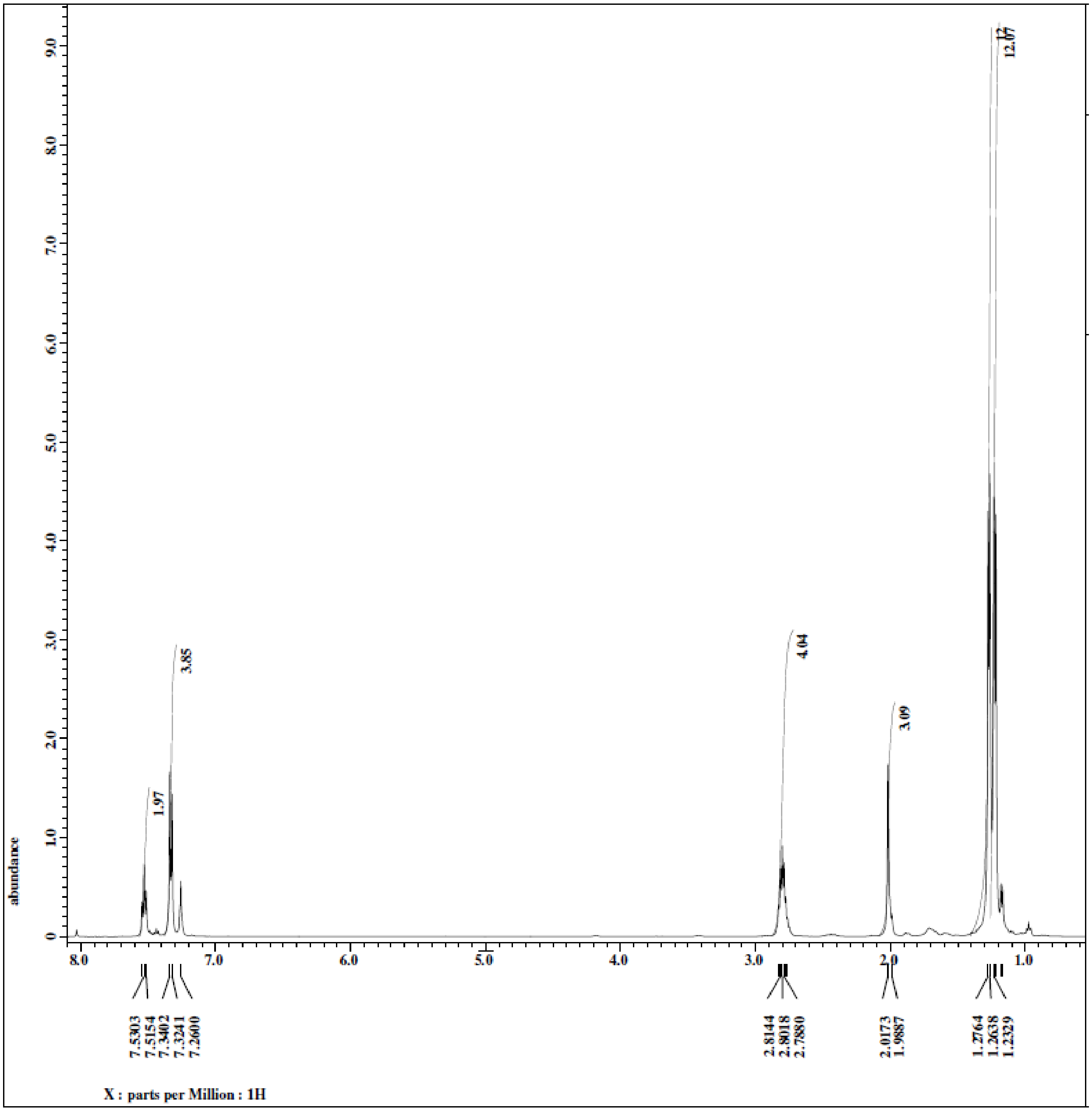

Figure S5. ^{1}H NMR spectrum of (Me-maloNHC$_{Dipp}$)HgCl (**1b**) in $CDCl_3$ at room temperature

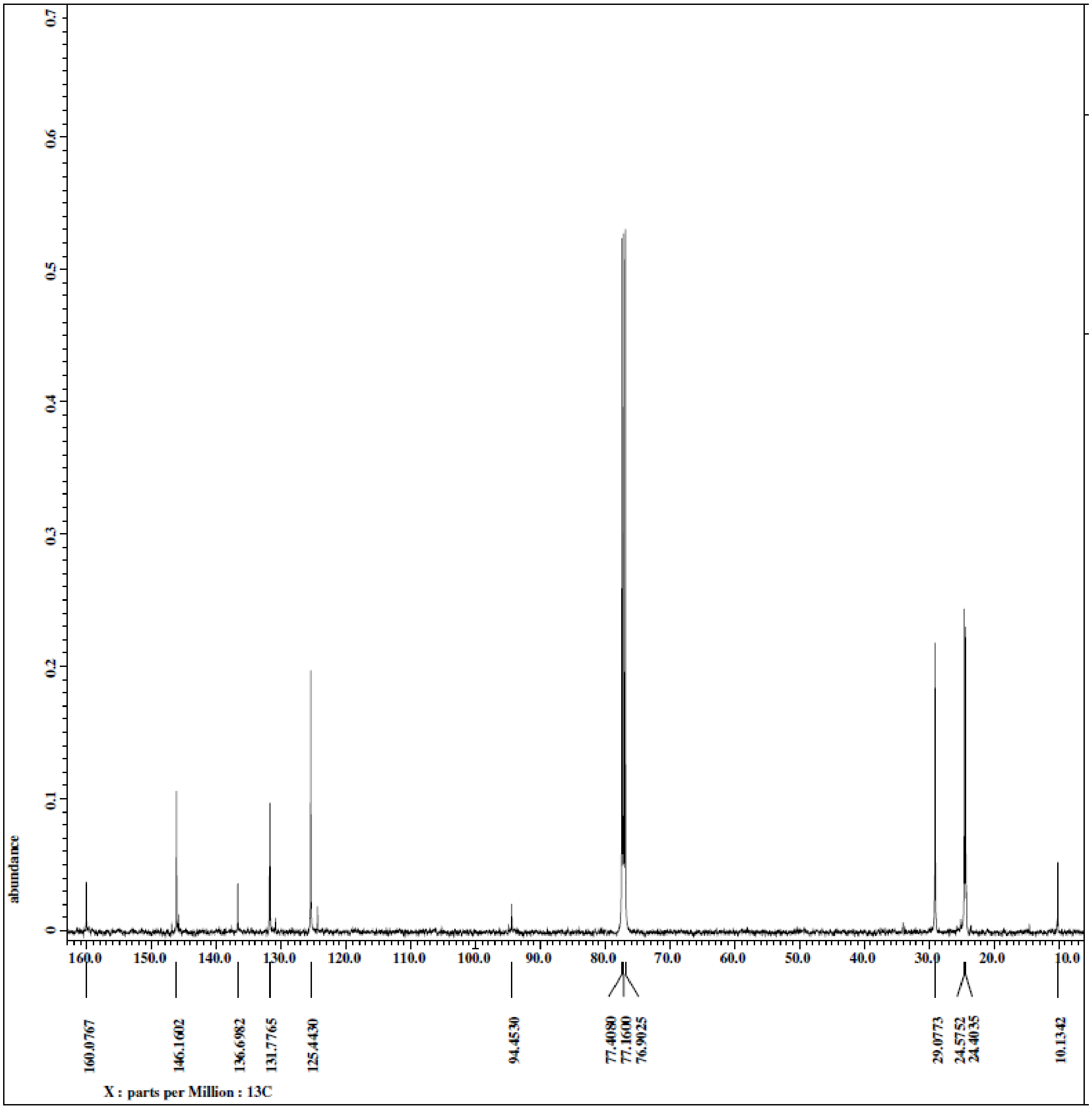

Figure S6. $^{13}C\{^{1}H\}$ NMR spectrum of (Me-maloNHC$_{Dipp}$)HgCl (**1b**) in $CDCl_3$ at room temperature

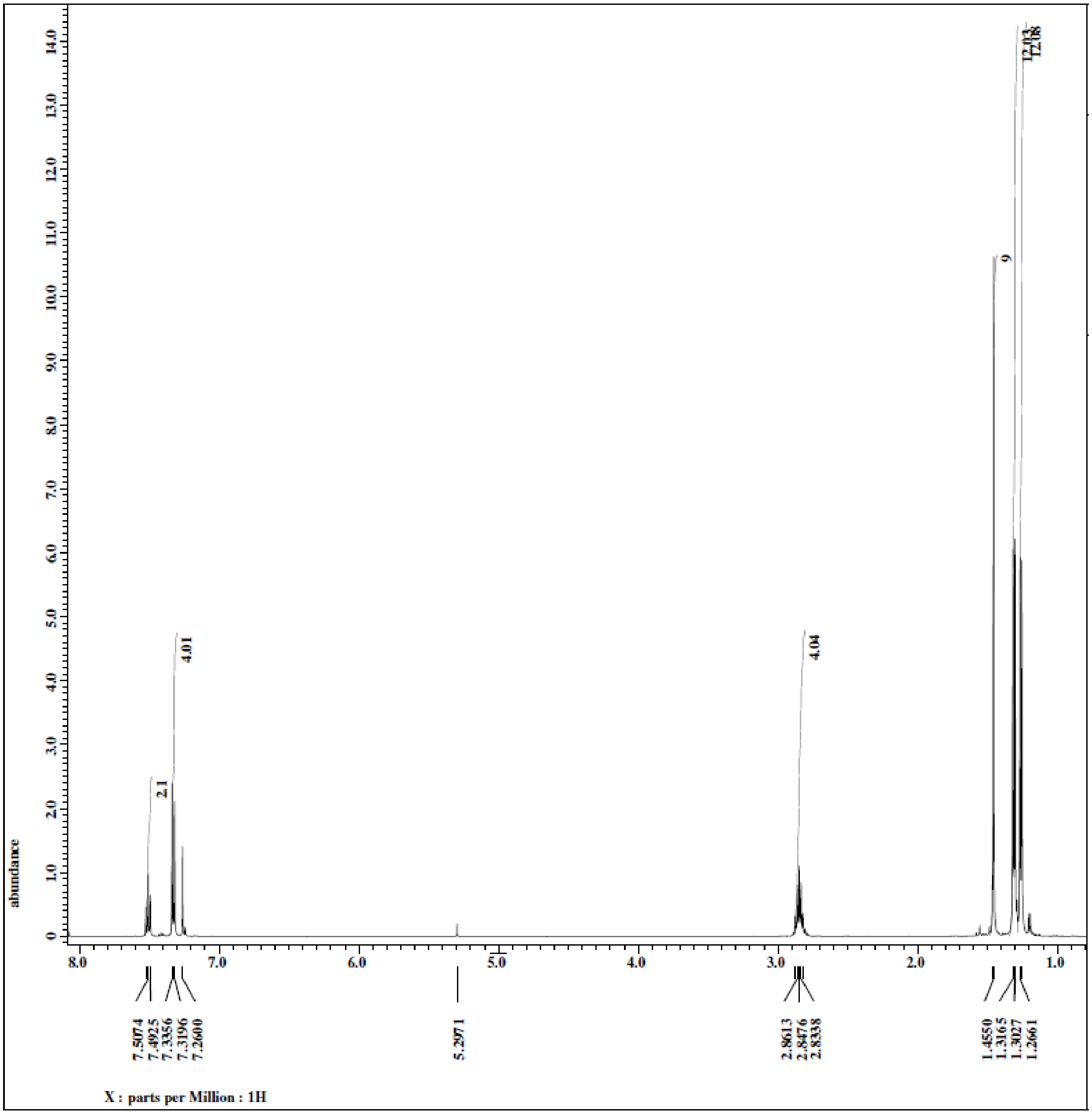

Figure S7. ^{1}H NMR spectrum of (*t*-Bu-maloNHC$_{Dipp}$)HgCl (**2b**) in $CDCl_3$ at room temperature

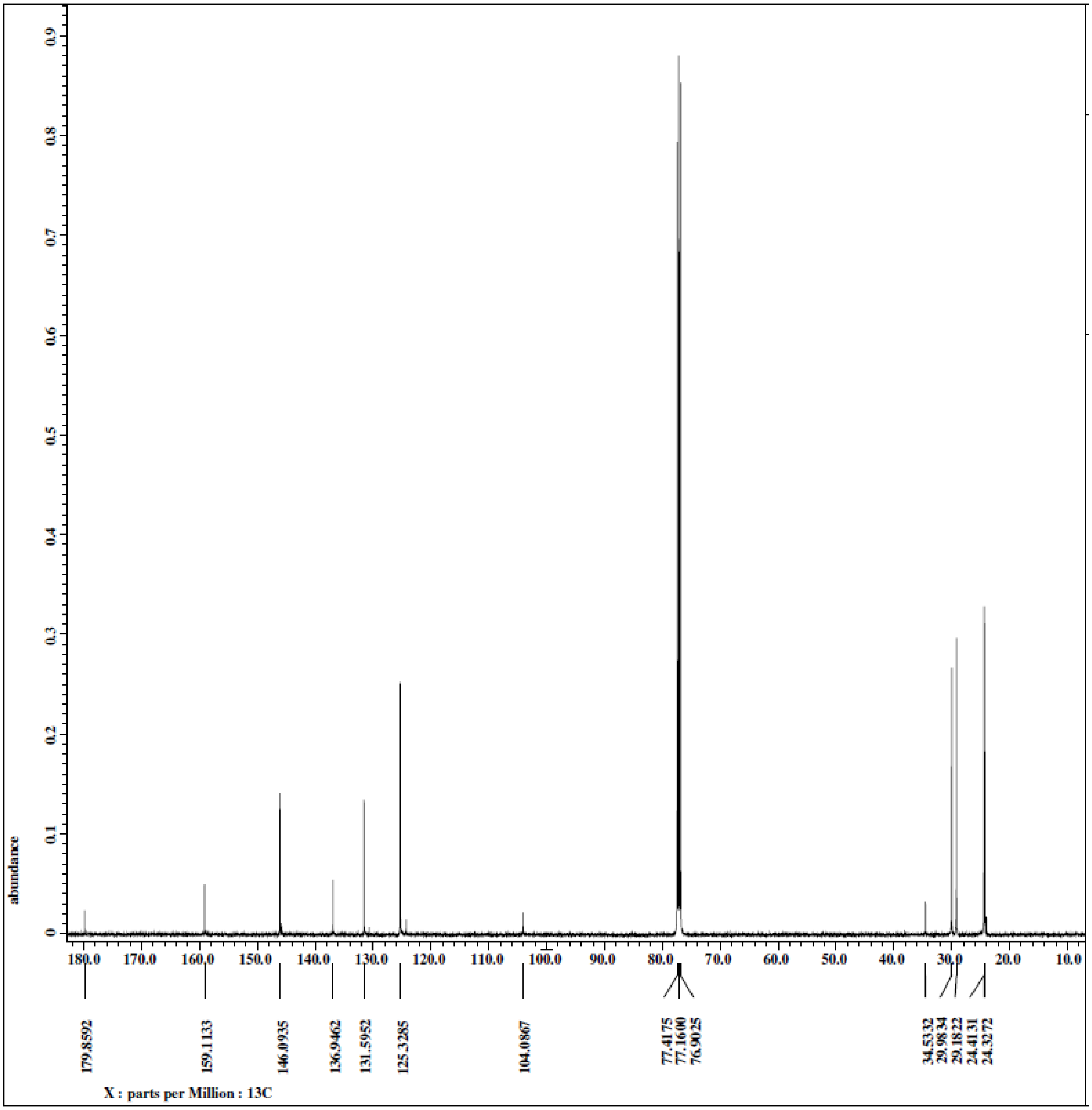

Figure S8. $^{13}C\{^{1}H\}$ NMR spectrum of (*t*-Bu-maloNHC$_{Dipp}$)HgCl (**2b**) in $CDCl_3$ at room temperature

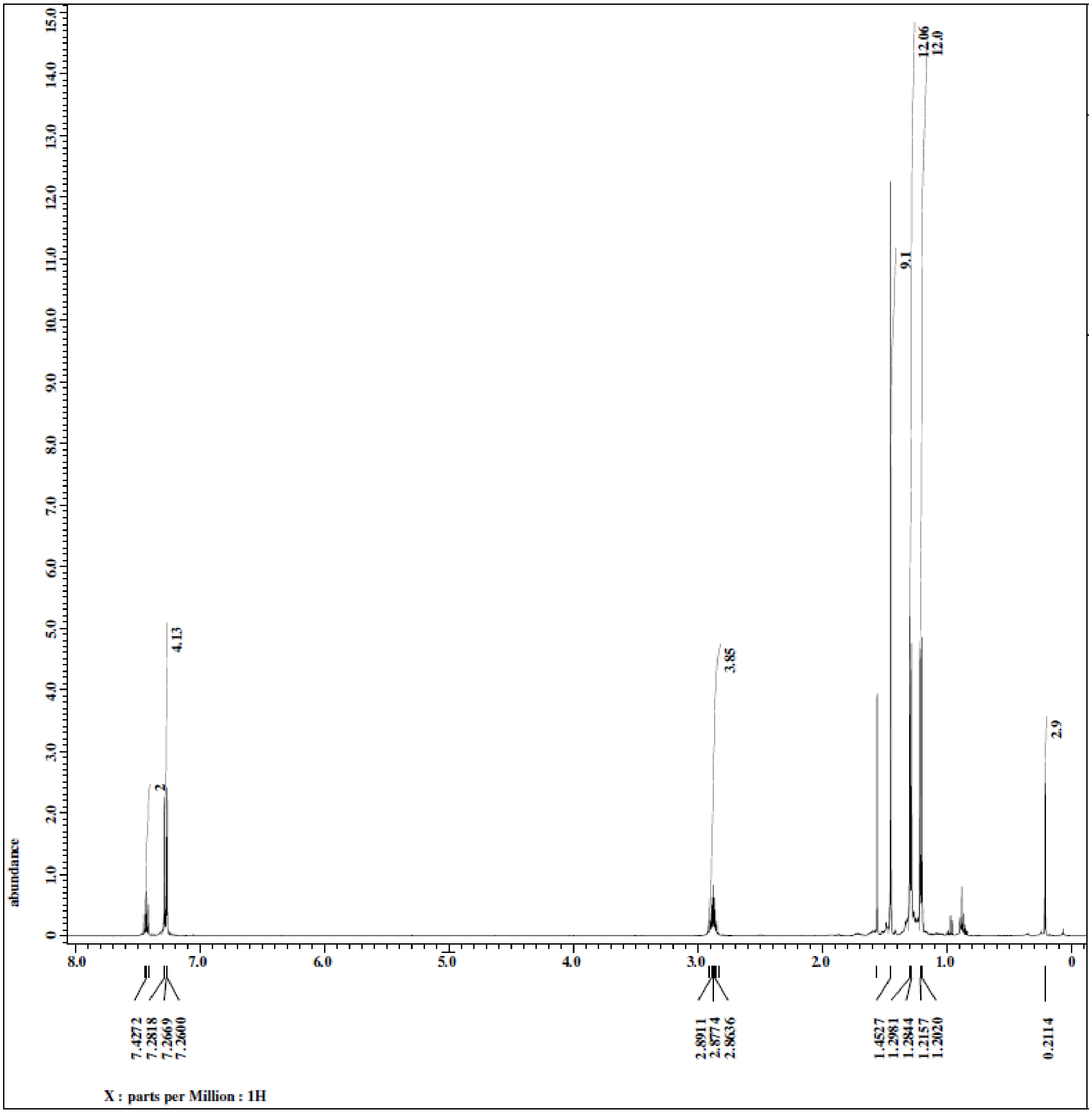

Figure S9. ^{1}H NMR spectrum of (*t*-Bu-maloNHC$_{Dipp}$)HgMe (**2c**) in $CDCl_3$ at room temperature

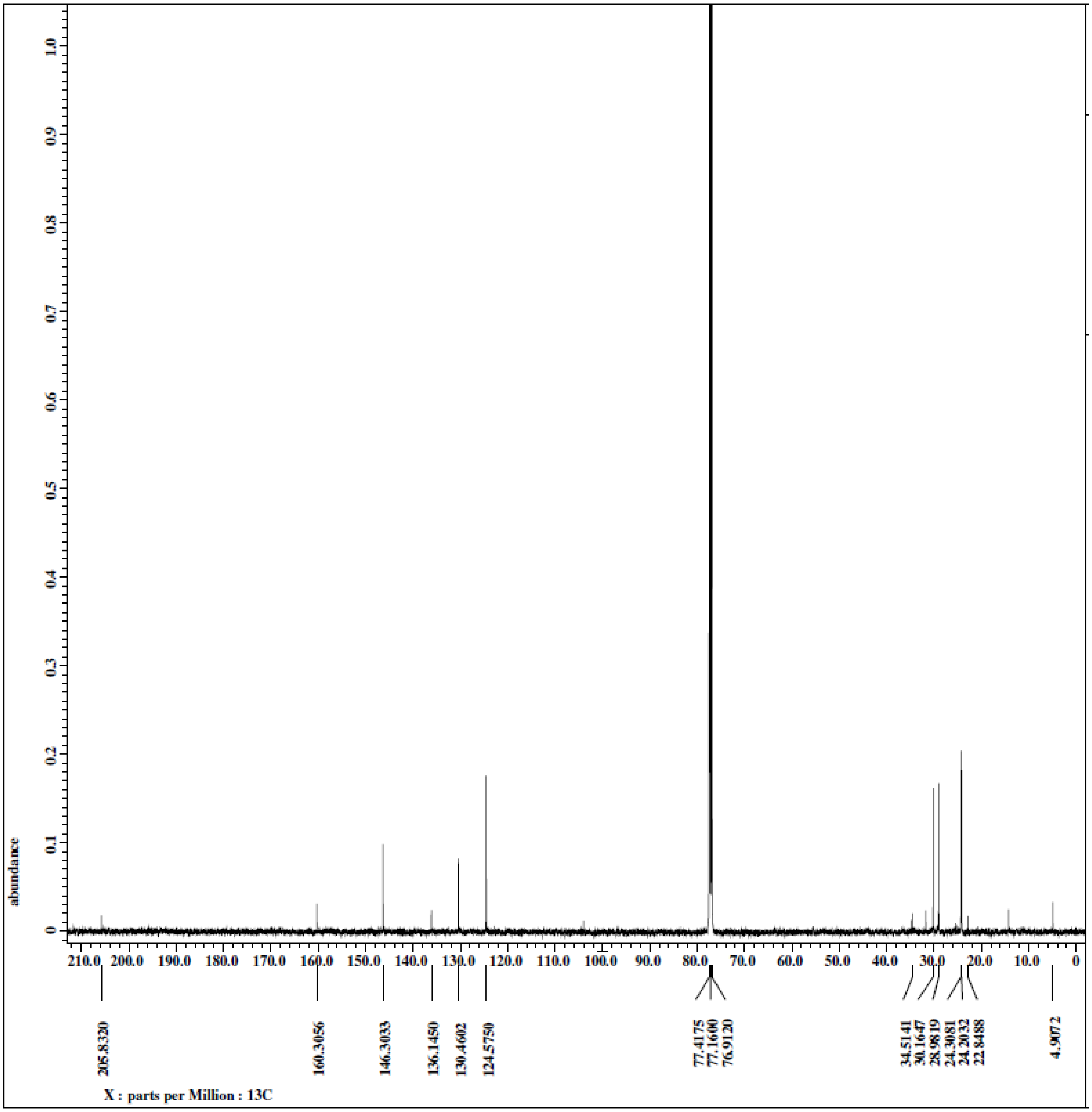

Figure S10. $^{13}C\{^{1}H\}$ NMR spectrum of (*t*-Bu-maloNHC$_{Dipp}$)HgMe (**2c**) in $CDCl_3$ at room temperature
